# Identifying Active Travel Behaviors in Challenging Environments Using GPS, Accelerometers, and Machine Learning Algorithms

**DOI:** 10.3389/fpubh.2014.00036

**Published:** 2014-04-22

**Authors:** Katherine Ellis, Suneeta Godbole, Simon Marshall, Gert Lanckriet, John Staudenmayer, Jacqueline Kerr

**Affiliations:** ^1^Department of Electrical and Computer Engineering, University of California San Diego, La Jolla, CA, USA; ^2^Department of Family and Preventive Medicine, University of California San Diego, La Jolla, CA, USA; ^3^Department of Mathematics and Statistics, University of Massachusetts Amherst, Amherst, MA, USA

**Keywords:** physical activity, random forest

## Abstract

**Background:** Active travel is an important area in physical activity research, but objective measurement of active travel is still difficult. Automated methods to measure travel behaviors will improve research in this area. In this paper, we present a supervised machine learning method for transportation mode prediction from global positioning system (GPS) and accelerometer data.

**Methods:** We collected a dataset of about 150 h of GPS and accelerometer data from two research assistants following a protocol of prescribed trips consisting of five activities: bicycling, riding in a vehicle, walking, sitting, and standing. We extracted 49 features from 1-min windows of this data. We compared the performance of several machine learning algorithms and chose a random forest algorithm to classify the transportation mode. We used a moving average output filter to smooth the output predictions over time.

**Results:** The random forest algorithm achieved 89.8% cross-validated accuracy on this dataset. Adding the moving average filter to smooth output predictions increased the cross-validated accuracy to 91.9%.

**Conclusion:** Machine learning methods are a viable approach for automating measurement of active travel, particularly for measuring travel activities that traditional accelerometer data processing methods misclassify, such as bicycling and vehicle travel.

## Introduction

Individual travel behavior has been important in transportation research and traffic planning for decades (1). More recently, active travel has also become a focus for public health (2). Studies of adults and children have shown that individuals who walk or bike for transportation, or use public transportation, accumulate more physical activity and are more likely to meet public health recommendations (3, 4). In some countries active travel has been related to obesity (5). These relationships, however, have been poorly studied because they are reliant on self-report data, which provide crude metrics (e.g., number of days vs. total minutes of active travel). The premise of active living research is that built environment can support more routine physical activity behaviors, and that if active travel is an equal choice compared to car travel, more people are likely to take advantage. Improvement in measurement of active travel will enable intervention studies trying to promote routine daily behaviors such as active travel.

Traditionally, travel behavior has been measured by travel and time use diaries or self-report surveys (6). Not only are these burdensome to participants, but also recall of events is often inaccurate and potentially biased (7–9). The emergence of lightweight, low cost, and accurate global positioning system (GPS) devices has enabled researchers to objectively track the location of individuals. However, while it is relatively straightforward to view and understand GPS data using Geographic Information System (GIS) packages, it would be extremely time consuming to do large-scale data analysis by manually interpreting each GPS track. In light of this, researchers began looking into automated ways of segmenting trips and identifying transportation mode from GPS data. Early studies of GPS data in transportation research focused on vehicle travel, simplifying the development of algorithms somewhat. More recently, multiple transportation modes have been studied, including active transportation.

There have been a variety of approaches to predicting transportation mode automatically from GPS data. These include heuristic rule-based algorithms, (10–13), fuzzy logic (14, 15), neural networks (16, 17), Bayesian models (18, 19), and decision trees (20, 21). These approaches rarely include non-travel activities (e.g., sitting or standing), and many incorporate map matching or GIS components, which are particularly useful in order to ascertain when a user is traveling on a public transit route. Physical activity researchers, however, may not have access to GIS data. In contrast, they are likely to include accelerometer data when assessing active travel (3, 4). Previous studies have shown that specific behaviors such as housework can be derived from accelerometer signals (22). These studies rarely include vehicle travel as an activity mode, are often performed in highly controlled lab settings, and mostly do not include GPS data that can inform trip mode.

Only a few studies have employed GPS and accelerometer data. Reddy et al. (23) use decision trees and Markov models to determine transportation mode on mobile phones, using both GPS and accelerometer data. They report 93% accuracy in predicting five activities (still, walking, running, bicycling, and vehicle). Troped et al. (24) also combine accelerometer and GPS data, but from standalone devices, using linear discriminant analysis to predict five activities (walking, running, bicycling, inline skating, and driving a car). They report 90% accuracy in predicting activities, but with a relatively small dataset of 712 min of data.

Many of these algorithms in the literature to date are only tested in ideal conditions or controlled environments, which may overestimate their accuracy. Conditions like instantaneous mode changes, cold start journeys, and trips in urban canyons can all interfere with signal detection and challenge the effectiveness of algorithms. Our novel contribution to the research includes a validation protocol that tested travel modes in multiple real world conditions, and collected a comprehensive dataset consisting of about 150 h of annotated data. We employed both GPS and accelerometer data, and used machine learning algorithms to identify transportation mode, using multiple features of both devices to inform the prediction model. We used a random forest algorithm, an efficient algorithm that to our knowledge has not previously been used to predict transportation modes from accelerometer and GPS data, although Lustrek and Kaluza (25) use a random forest algorithm for activity recognition from 12 small infrared motion tags placed on a user’s body and Casale et al. (26) use random forests for physical activity recognition from accelerometer data.

## Material and Methods

### Data collection procedures

Two trained research assistants in San Diego followed a data collection protocol. They collected data under varying conditions (i.e., open space vs. urban, indoor vs. outdoor), for a variety of transportation modes (walking, driving, etc.). The researchers wore an Actigraph GT3X+ accelerometer on the hip and 12 devices attached to 2 wooden boards (9″ × 12″) carried in a backpack. Board-mounted devices were attached with Velcro to ensure device antennae were all aligned in the same way. The board contained 12 devices, each with different settings: 2 different GPS models, at 3 different epochs with either warm or cold start conditions (i.e., device on and signal obtained or device switched on immediately before travel and no signal established). The GPS device we used in this analysis was a Qstarz BT1000X set to collect data at 15 s epochs using warm starts. The accelerometer device collected data at 30 Hz on three axes. The researchers followed a set protocol of trips, pauses, and locations. There were at least 4 example trips per condition resulting in over 500 trips. The researchers kept a log of each trip, location, and condition settings and noted start times for each event. A research manager reviewed files on a daily basis and reallocated trips that were not successfully completed or noted. (The full study protocol is available from the authors). All the data are considered at the trip level, the individual characteristics of the two data collectors were not included.

Table 1 outlines the different conditions under which GPS and accelerometer data were collected following the protocol. Table 2 reports the total minutes of data collected in each transportations mode, and includes periods of time between the prescribed trips during data collection.

**Table 1:** **Prescribed trip parameters for data collection**.

Condition	Description	Number of trips
**First level environment variation**		511 (total)
Urban canyon	Downtown areas with high rise buildings that interfere with GPS signal	259
Open space	Areas without high rise buildings where GPS signal connectivity is high	252
**Second level transition and location variation**	
Continuous	A continuous connection between transportation modes, e.g., stop car and passenger started walking immediately. Most naturally occurring trip transitions are continuous	142
Pause	A 2-min pause between transportation modes. Pauses enable trip ends to be detected more easily	192
Indoor/outdoor transition	Stationary periods indoors and outdoors were tested, as well as transitions between indoors and outdoors including transitions every 30 s from indoor to outdoor environments. The Qstarz device allows collection of satellite ratios, which can help to detect indoor vs. outdoor locations.	22
**Building types**	
Full/partial signal buildings	Single story buildings with large windows, wooden roofs, and open courtyards	12
Blocked signal	Multistory buildings, underground garages	12

**Table 2:** **Minutes of data collected for each transportation mode**.

	Minutes of data	Percent of total (%)
Bike	857.5	10
Bus	632.3	7
Car	2063.0	23
Sit	849.5	9
Stand	1631.3	18
Walk	2490.3	28
Unclassified	464.0	5
Total	8987.8	100

### Classification pipeline

**Figure 1:**
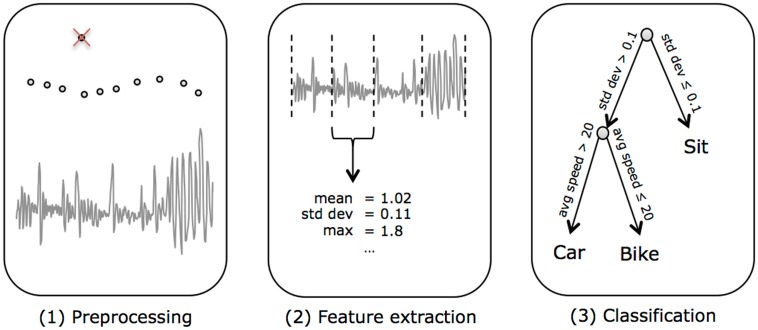
**The classification pipeline**. (1) We started from raw sensor data, which was split into 1-min windows. (2) Features were extracted from each window of data. (3) Then the features from each window were classified into transportation modes.

Each step in the data classification pipeline is detailed in the following sections. Raw data were first preprocessed to remove common GPS errors. The data were then split into 1-min windows and feature vectors were extracted from each window. Each window was then classified using a machine learning algorithm. Figure 1 shows an overview of the classification pipeline, which starts from raw sensor data and produces classifications for each minute of data.

### Data preprocessing

Data smoothing typically occurs before data are employed in a machine learning environment. GPS data were first processed by the Personal Activity Location Measurement System (PALMS) (27). PALMS filters spurious data points and smoothes out common GPS interference patterns. PALMS uses a set of simple filters to remove invalid coordinates and reduce data volume. The filters include removal of points with excess speed; large changes in elevation or very small changes in distance between consecutive points; and scatter caused by interference from buildings. For periods of signal loss PALMS imputes the previous valid coordinates (28).

### Trip segmentation

Many previous approaches to travel mode classification do a first step of segmenting the GPS stream into cohesive trips consisting of a single travel mode (10, 12, 14). This segmentation is usually done based on simple rules that make some assumptions about the way people travel – for example, people always walk in between trips of different modes, or are always stationary for a certain length of time in between trips. However, these assumptions may not hold in the real world – in fact the data in this study were collected in order to explicitly violate these assumptions. Therefore, instead of segmenting the data into trips, in our method we individually classified each minute of data with a travel mode. These predictions are then smoothed with a simple moving average filter that encourages consecutive minutes of data to be classified with the same mode. Trips can then be easily defined by grouping consecutive minutes classified with the same travel mode. This approach prevents the use of heuristics that enforce a specific ordering of transportation modes.

### Feature extraction

Most machine learning algorithms require inputs to be example data points consisting of real numeric data. These inputs are called feature (or attribute) vectors. Our input data consist of streams of accelerometer and GPS data. The feature extraction step is the process of transforming these data streams into feature vectors that capture relevant and predictive information. We used a sliding window to break the data stream into 1-min windows of accelerometer and GPS data, each with a corresponding transportation mode label. If the window spanned multiple different transportation modes, or an unlabeled segment of data, we left it unlabeled. Consecutive windows overlap by 30 s. We summarized each 1-min window by computing a feature vector consisting of descriptive statistics of the data in that window (e.g., average speed, correlation between accelerometer axes, etc.). We normalized the features to have mean zero and standard deviation one, to account for the scale difference between features (i.e., acceleration measurement is between ±6 G, while GPS speed is commonly above 40 mph). We computed a 49-dimensional feature vector for each minute of data, consisting of 43 acceleration features and 6 GPS features. Using a 1-min window resulted in 17,916 example minutes in our dataset, 14,307 of which had valid labels.

#### Acceleration features

An 1-min window of acceleration measurements contains *T* = 60s × 30 Hz × 3 axes = 5400 samples of acceleration measurements along the *x, y*, and *z* axes, which we represent as a matrix,

$$A=\left[\begin{array}{cccc} a_{1,x} & a_{2,x} & \ldots & a_{T,x}\\ a_{1,y} & a_{2,y} & \ldots & a_{T,y}\\ a_{1,z} & a_{2,z} & \ldots & a_{T,z} \end{array}\right].$$

**Figure 2:**
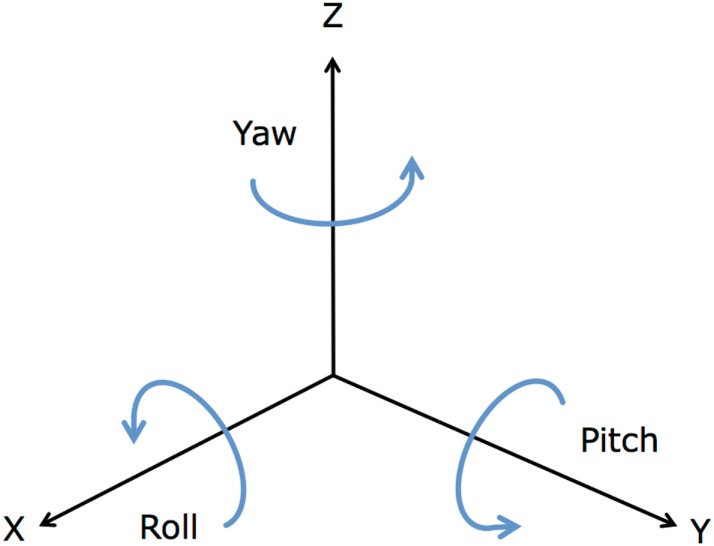
**Average roll, pitch, and yaw angles**.

The data from this window are condensed to 43 acceleration features. Most features are computed from the vector magnitude of the 3-axis acceleration,

$$a_{t}=\sqrt{a_{t,x}^{2}+a_{t,y}^{2}+a_{t,z}^{2}}\;,$$

although some features (for example, correlations between axes) are computed differently. We compute the following features:

- *Basic descriptive statistics* computed from the vector magnitudes a_1:T_: mean, standard deviation, 25th and 75th percentiles, minimum, and maximum. These are features commonly used in previous work predicting physical activity from accelerometers (21, 23, 24).
- *Skewness and Kurtosis*, descriptive statistics derived from the third and forth moments of the data distribution, that measure the asymmetry and peakedness, respectively, of the distribution of accelerometer magnitudes in a minute.
- *Autocorrelation* of the vector magnitude with 1-s lag (17).
- *Correlations* between each pair of axes of the accelerometer (i.e., *x*–*y* correlation, *x*–*z* correlation, and *y*–*z* correlation).
- *Entropy*, a measurement of the randomness of the distribution of accelerometer magnitudes.
- *Angular features*, to provide information about the orientation of the accelerometer in space. The roll, pitch, and yaw are measurements used in aeronautics to describe the rotation of an aircraft, and are calculated by:
  $$\begin{array}{llll} \text{average roll} & =\frac{1}{T}\sum_{t-1}^{T}\tan^{-1}\left(a_{t,y},a_{t,z}\right)\; & & \;\\ \text{average pitch} & =\frac{1}{T}\sum_{t-1}^{T}\tan^{-1}\left(a_{t,x},a_{t,z}\right)\; & & \;\\ \text{average yaw} & =\frac{1}{T}\sum_{t-1}^{T}\tan^{-1}\left(a_{t,y},a_{t,x}\right)\; & & \; \end{array}$$
  Figure 2 shows these angles on the coordinate axes.
- *Principal direction of motion*, obtained via Eigen-decomposition of the acceleration covariance matrix, AA^T^. In particular, we determined the principal direction of motion by taking the eigenvector ν of AA^T^ with corresponding maximal eigenvalue – this corresponds to the direction with maximum variation.
- *Autoregressive coefficients*: we model the acceleration vector magnitude by an autoregressive model of order *p* = 5,
  $$a_{t}=c_{0}+\sum_{i=1}^{p}c_{i}x_{t-i}+\varepsilon_{t},$$
  where c_0_, … ,c_p_ are the model coefficients, and ε*_t_* is white noise.
- *Fast Fourier Transform (FFT) coefficients*: the FFT decomposes the signal, e.g., the time series of acceleration measurements, into components of different frequencies, transforming a time domain signal *a_t_* to a frequency domain signal *A_f_*. From the FFT, we computed the power spectrum ${\left|A_{f}\right|}^{2}$ for frequencies *f*  = 1–15 Hz.
- *Total power* in the signal from 0 to 15 Hz.
- *Dominant frequency*, the frequency corresponding to maximal power in the power spectrum, and corresponding power.

#### GPS features

We obtained six features from the GPS device: average speed, average number of satellites used and in view, average signal-to-noise ratio (SNR) of satellites used and in view, and net distance traveled in the minute. These 6 features were appended to the 43 acceleration features to obtain the 49-dimensional feature vector that describes each minute of data.

### Machine learning methods

Our goal in this work is to use supervised machine learning methods to predict transportation mode from streams of accelerometer and GPS data. The term “supervised” refers to the fact that we make use of a training data set containing examples of accelerometer and GPS data with known corresponding labels (i.e., transportation mode). A supervised learning algorithm analyzes the training data and produces an inferred function, or classifier, which maps a data point (represented by its feature vector), to a label. This is in contrast to “unsupervised” learning algorithms, which use data without labels to identify some sort of underlying structure in the data (i.e., clustering methods). A labeled training dataset is made up of pairs of feature vectors and labels. There are a wide variety of classifier functions and learning algorithms that could be used, and we tested several of the more popular choices.

#### Classification

We tested several well-known machine learning algorithms to classify transportation mode: *k*-nearest neighbor (*k*NN), support vector machines (SVM), naive Bayes, decision trees, and random forests. Many software packages exist that implement these algorithms and allow them to be used as a “black box” to perform classification of data. Of these algorithms, the random forest algorithm, which is an ensemble method based on decision trees, produced the highest accuracy, and thus the remainder of the analysis in this paper will focus on the random forest algorithm. A decision tree is a type of classifier that consists of leaves representing class labels and branches representing conjunctions of features that lead to those class labels. For a test data point, the class label is found by traversing the tree according to the conjunctions in the branches of the tree, and when a leaf is reached the label in that leaf is assigned to the data point. The training phase of the algorithm consists of building the decision tree, i.e., learning the branches that lead to a tree that correctly classifies as many examples in the training data set as possible. A random forest combines the outputs of multiple randomized decision trees. Shotton et al. (29) use random forests to do human pose recognition for the Xbox Kinect. To learn each decision tree, we chose a random subset of 10,000 training examples (at 1-min epoch) and a random subset of 25 features. We learned 100 of these randomized decision trees. To classify a given test example, we traversed each tree until we arrived at a leaf node. Each leaf node has a probability score for each transportation mode, according to the ratio of training examples of each transportation mode that land in that node. We summed these probability scores in the final leaf node over the 100 trees, and chose the transportation mode with highest probability for our test example. We choose the parameters for our classification algorithms (i.e., number of trees to use) using a held-out day of data that was not included in the final cross-validation results. For the decision trees, we set the minimum number of examples in a leaf to be 10. For the *k*NN algorithm *k* was chosen to be 3 and for SVM the regularization parameter was chosen to be 10.

### Moving average output filter

We filter the output predictions from the random forest classifier with a simple moving average filter. This filter looks at the predictions made in the 2 min previous to and 2 min following the minute in question, and outputs the mode that is predicted the highest number of times. If there is a tie, it outputs either the prediction from the current minute (if this is one of the tied predictions), or the prediction from the earlier of the tied minutes. This prevents rapid switching between different modes and encourages successive predictions to belong to the same travel mode.

## Results

We tested the performance of each machine learning algorithm by performing leave-one-day-out cross-validation. This corresponds to a realistic setting in which the algorithm would be used – the data from within a trip are never used as training data to classify another piece of data from that same trip. Using standard *k*-fold cross validation that allows data from the same trip in the test and training set produces artificially high accuracy scores.

In addition to overall accuracy, we evaluated the performance of each classifier using precision, recall, and *F*-score. Precision measures the proportion of predicted examples of an activity type that are correct. Precision (*P*) is calculated as *P* = TP/(TP + FP), where TP is the number of true positives, and FP is the number of false positives. Recall measures the proportion of true examples of an activity type that are correctly identified (also called sensitivity). Recall (*R*) is calculated as *R* = TP/(TP + FN), where TP is the number of true positives, and FN is the number of false negatives. *F*-score is a measure of accuracy, and is computed as the harmonic mean of precision and recall, *F*-score = 2*PR*/(*P* + *R*). These metrics provide detailed information about how the algorithm performs on each class. The *F*-scores obtained from each algorithm are shown in Table 3. The random forest algorithm showed the highest performance, with an overall accuracy of 89.8%.

**Table 3:** **Performance results for various classifiers (without output filtering)**.

	*F*-score							Overall accuracy (%)
	Bike	Bus	Car	Sit	Stand	Walk	Average	
*k*NN	0.924	0.585	0.855	0.682	0.829	0.955	0.805	86.2
Naïve Bayes	0.872	0.220	0.824	0.503	0.484	0.920	0.637	74.2
SVM	0.962	0.609	0.884	0.724	0.833	0.954	0.828	87.7
Decision tree	0.922	0.537	0.846	0.674	0.792	0.936	0.785	83.6
Random forest	0.971	0.601	0.888	0.778	0.855	0.962	0.843	89.8

**Figure 3:**
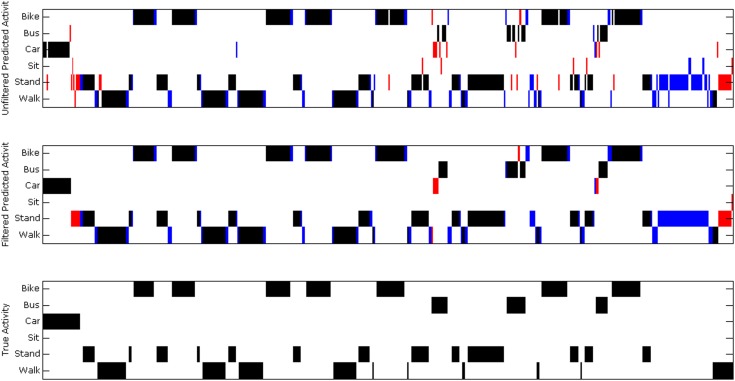
**Example output for 1 day of data**. We plot the activity mode vs. time for 1 day of data. In the top plot, we show the activity mode predicted by the random forest algorithm. In the middle plot, we show the smoothed predictions output by the moving average filter. In the bottom plot, we show the ground truth annotations for this day. Minutes in black were correctly classified, minutes in red were misclassified, and minutes in blue had no ground truth annotation with which to compare.

Using the moving average output filter to smooth predictions significantly improved results, leading to an average precision of 0.900, average recall of 0.882, and overall accuracy of 91.9%. Table 4 reports the precision, and recall scores before and after output filtering. Figure 3 shows an example day of data, before and after smoothing, compared to the ground truth annotations for the day. Table 5 shows the confusion matrix for the smoothed random forest classifier, which reports the number of test examples classified in each transportation mode. The diagonal emboldened numbers represent the correct predictions by mode.

**Table 4:** **Precision (*P*) and recall (*R*) results for the random forest classifier with and without output filtering**.

		Bike	Bus	Car	Sit	Stand	Walk	Average	Overall accuracy (%)
No output filter	*P*	0.982	0.795	0.880	0.779	0.844	0.968	0.874	89.8
	*R*	0.979	0.545	0.934	0.832	0.880	0.945	0.853	
Output filter	*P*	0.985	0.860	0.910	0.807	0.878	0.962	0.900	91.9
	*R*	0.976	0.701	0.952	0.821	0.871	0.970	0.882	

**Table 5:** **Confusion matrix for the random forest classifier with output filtering**.

	Bike	Bus	Car	Sit	Stand	Walk
Bike	1526	18	4	0	8	3
Bus	2	611	409	54	34	11
Car	2	127	3563	42	69	13
Sit	0	1	44	1232	186	17
Stand	5	8	8	228	2546	97
Walk	19	4	22	26	174	4228

*Rows represent number of examples of true activities; columns represent number of examples of predicted activities. Entries along the diagonal indicate correct predictions*.

### Importance of features

In order to gain insight into the usefulness of each feature, we computed an importance score. This score was computed by summing the changes in the training error each time a feature was used to create a new branch in a decision tree, and averaged over each tree in the random forest. We then normalize the scores to sum 1 over all the features. Table 6 shows the top 15 features according to this importance score.

**Table 6:** **Top 15 most informative features**.

	Score
Standard deviation	0.251
Average speed	0.147
Net distance covered	0.085
Power at dominant frequency	0.082
Autocorrelation	0.061
Average yaw	0.044
Average roll	0.039
Minimum	0.034
FFT 4 Hz	0.029
FFT 3 Hz	0.022
Correlation between *x* and *y* axes	0.018
Maximum	0.018
25th Percentile	0.013
Total power	0.013
Average SNR used	0.013

We computed an importance score for each feature and ranked the features according to this score

## Discussion

This study employed GPS and accelerometer data to identify transportation mode for trips collected in varying environmental conditions. Machine learning methods were employed to extract features of the data stream and build an algorithm to predict transportation mode. The algorithm was shown to have over 90% accuracy on leave-one-day-out cross-validation.

Few previous studies have employed GPS and accelerometer data [for example, Reddy et al. (23)] and machine learning techniques to predict transportation mode. Our study demonstrated similar accuracy rates as Reddy et al., but deployed the devices over a larger number of trips, which varied by environmental features, thereby providing a more challenging test of the algorithms. Another difference was the Reddy study employed smart phones whereas we used research-grade accelerometers and GPS devices mounted at specific locations. Although smartphones are becoming ubiquitous technologies for continuous sensing of geolocation and acceleration data, they are limited because of competing power demands of the phone or other functions (e.g., there must be sufficient power for the phone to make calls, text, read email, surf the web, etc., with limited interruption to other sensor inputs). It is also unclear if the integrity of smartphone sensor data relies on the phone being in a fixed position (i.e., the person keeping the phone in the same position all day). Although recent studies have attempted to circumvent these issues, solutions appear largely experimental or prototypical (30, 31). Importantly, there have been few studies in the transportation literature that have included accelerometer data to improve prediction of transportation mode. Previous studies have employed machine learning techniques on GPS data alone, but the diversity of the training data was unknown.

The analysis of the importance of each feature demonstrated that the accelerometer data contribute additional predictive power above the GPS data. The feature with highest importance was the standard deviation of the acceleration, which captures information about the signal variability. Stationary sitting and standing should consistently produce low speeds and accelerations, while bus and car have a much wider range of possible values. The feature with the second highest importance score was the speed from the GPS, which differentiates fairly well between activities with very different average speeds, such as vehicle vs. walking and sitting or standing. Although vehicle speeds are often higher than active transportation modes, in downtown corridors vehicle speeds can be slow including periods where the vehicle has stopped altogether, such as at a traffic light. These movements may mimic that of walking or biking. The net distance covered feature is computed from the GPS data, by simply computing the distance between the first and last latitude and longitude points in a data window. It is helpful in determining whether substantial forward progress was made during the minute, which is helpful, for example, in differentiating walking from standing. Another important feature was the power at the maximum frequency – this measures whether the acceleration signal has a strong dominant frequency, which is exhibited in signals that are highly periodic, such as walking or bicycling. Walking in particular has a very consistent dominant frequency between 3 and 4 Hz, which accounts for the high importance of the 3–4 Hz feature. Another interesting feature is the average roll, which provides information about the angle with which the device is positioned. Since the device is firmly affixed to the participant’s hip can provide information about whether the subject is bent at the hip, i.e., sitting vs. standing.

This study demonstrated that under varied environmental conditions known to affect GPS signal, transportation modes could be detected with high accuracy. Although the accelerometer and GPS data were collected from separate devices, most mobile phones now collect GPS and accelerometer data. Additional studies could investigate algorithms applied to such mobile phone data. While mobile phone implementation of this study would lose some standardization of device placement (i.e., subjects could hold the phone wherever they like), which may make the angular features in particular less helpful, these features may instead provide some information about device placement, which can be correlated with transportation mode. Studies in free-living populations should also be conducted to confirm the generalizability of these algorithms. New sensors such as the SenseCam that provides accompanying image data may make this possible (32).

Limitations of this study include the small sample of subjects (*n* = 2; although each subject provided over 200 trips). When participants are performing prescribed trips, there should be less variation between participants than in a free-living scenario. In particular, the GPS data of two participants performing the same prescribed trip should look very similar. For this reason we chose to focus on collecting data from a wide variety of environments rather than a wide variety of participants. Validating methods on a controlled dataset is a first step before applying them to larger free-living dataset, which is the subject of future work. Additional limitations are that the data were only collected in one county (although the environments purposefully varied), and application of the algorithms to only one GPS model. Additionally, we only tested our algorithms on windows of data that fell completely within a bout of certain transportation mode – i.e., we did not include windows containing transitions between activities. This was done because evaluating the performance of our algorithm on these windows that have no single ground truth label is not straightforward. Future work should determine a metric for assessing the performance of the algorithm on these split class windows. One of the main sources of error in our classification was confusion between bus and car transportation modes, which is to be expected since the two modes are so similar. Including information from GIS systems, such as the proximity of public transportation routes, has the potential of greatly improving the distinction between these two modes, and we plan on addressing this in future work. Additionally, future work will test the effect of warm vs. cold start GPS data.

## Author Contributions

Katherine Ellis developed and implemented the algorithm, analyzed results, and wrote the manuscripts. Suneeta Godbole coordinated data collection and data management and critically revised the manuscript. Simon Marshall and Jacqueline Kerr developed the study design, coordinated data collection, and critically revised the manuscript. Gert Lanckriet and John Staudenmayer advised on algorithms and critically revised the manuscript.

## Conflict of Interest Statement

The authors declare that the research was conducted in the absence of any commercial or financial relationships that could be construed as a potential conflict of interest.
